# Toward an integrated risk paradigm: personalizing antithrombotic therapy for coronary heart disease with gastrointestinal bleeding

**DOI:** 10.3389/fcvm.2026.1782409

**Published:** 2026-04-14

**Authors:** Ai-lian Dong, Xi Chen, Yuan-ting Qu, Qi-han Huang, Si-jian Feng

**Affiliations:** 1 Department of Gastroenterology, Hongqi Hospital Affiliated to Mudanjiang Medical University, Mudanjiang, China; 2 Department of Cardiology, Hongqi Hospital Affiliated to Mudanjiang Medical University, Mudanjiang, China

**Keywords:** antiplatelet therapy, antithrombotic therapy, coronary artery disease, gastrointestinal bleeding, multimorbidity, personalized medicine, risk integration

## Abstract

The management of antithrombotic therapy in coronary artery disease has long been constrained by the tension between preventing ischemia and provoking bleeding. Current approaches often treat these risks as competing priorities, leading to reactive and unstable treatment plans. This perspective article argues for a fundamental shift toward a risk-integration paradigm, which re-conceptualizes ischemic and bleeding risks as interconnected components of a unified, dynamic patient profile. By adopting a framework of continuous assessment, mechanism-based therapy, phased management, and shared decision-making, this paradigm offers a systematic and individualized strategy for optimizing long-term clinical outcomes.

## 1 Introduction

For patients with coronary artery disease (CAD), particularly after percutaneous coronary intervention (PCI) or with concurrent atrial fibrillation, intensive antithrombotic therapy is essential to prevent stent thrombosis and stroke (1, 2). However, such regimens—often involving dual antiplatelet therapy or combined anticoagulation—markedly increase the risk of gastrointestinal bleeding (GIB) (1, 3, 4). GIB events are not only serious clinical endpoints in themselves, frequently leading to hospitalization and transfusion, but also commonly force the interruption of antithrombotic treatment (5). This interruption can precipitate recurrent ischemic events, creating a vicious cycle of bleeding and ischemia.

This precarious balance places clinicians in a persistent therapeutic dilemma. Current guidelines recommend risk stratification tools such as PRECISE-DAPT and HAS-BLED to individually assess bleeding and ischemic risks (6, 7). In practice, however, these tools are frequently applied in a parallel yet disconnected manner, offering fragmented guidance that can result in treatment plans alternating between excessive and insufficient antithrombotic intensity (8). This approach reflects an underlying “risk-confrontation” paradigm, wherein managing one risk (ischemia) often inadvertently exacerbates the other (bleeding), without integrating both into a unified clinical assessment (9).

**Figure 1:**
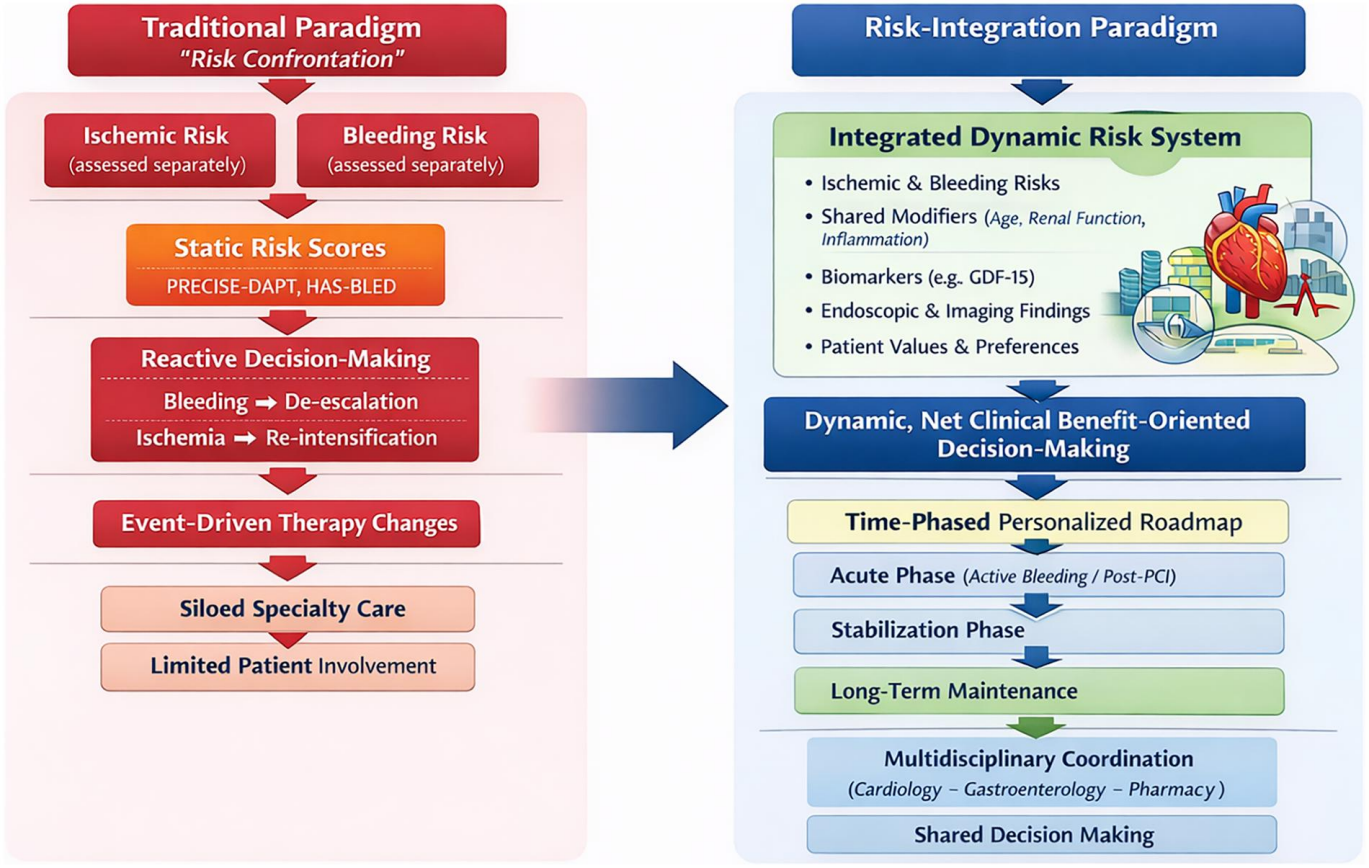
From risk confrontation to risk integration in antithrombotic therapy.

We therefore argue that for CAD patients at risk of GIB, the conventional “risk-confrontation” model is insufficient (10). A fundamental shift toward a “risk-integration” paradigm is urgently needed (Figure 1). This new framework does not view ischemic and bleeding risks as opposing forces to be traded off, but rather as interrelated components of a single clinical profile that should be managed cohesively. It emphasizes systematic and dynamic risk assessment, continuous monitoring, and personalized therapeutic strategies designed to minimize overall risk.

**Figure 2:**
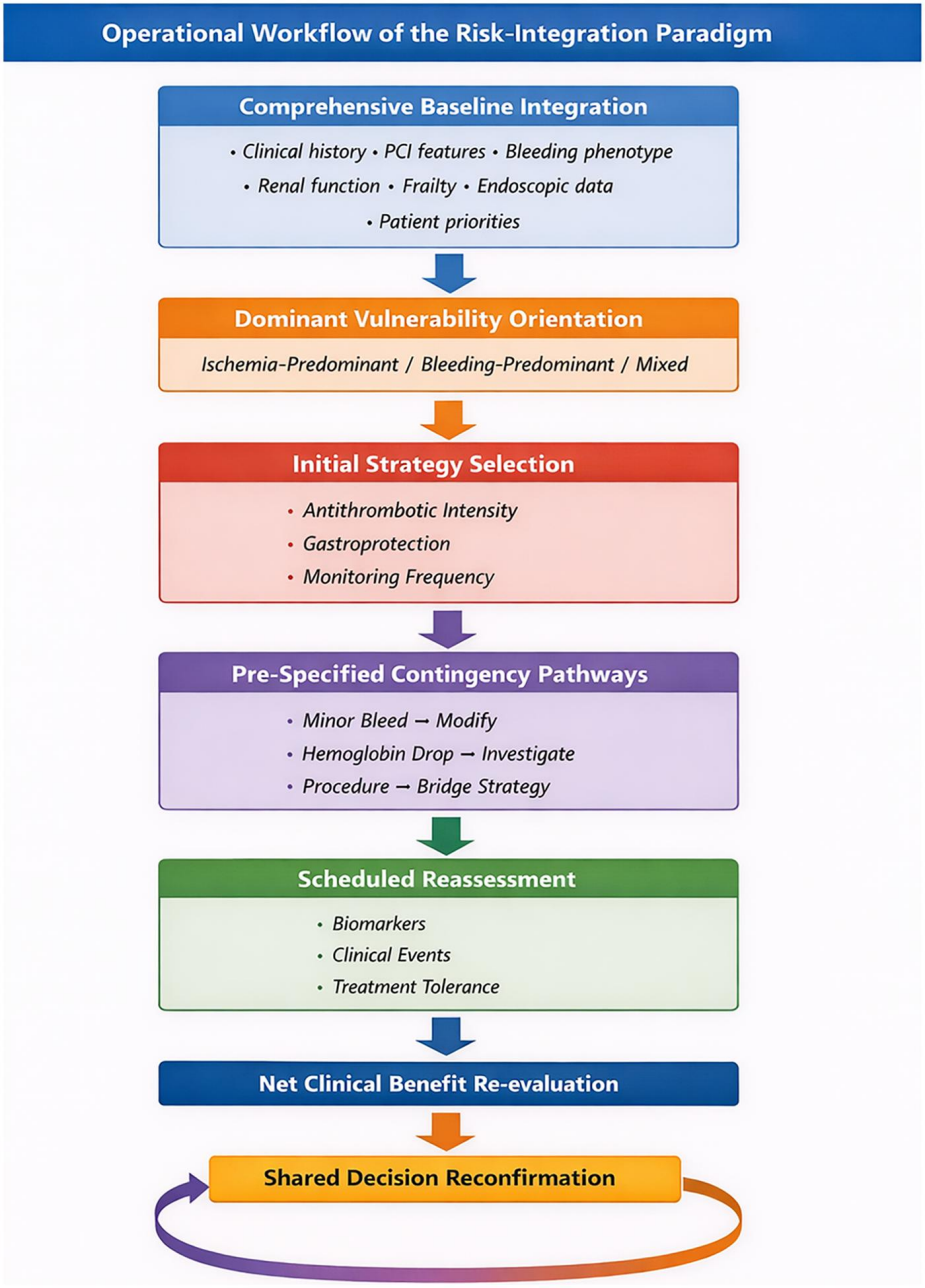
Operational workflow of the risk-integration paradigm.

This perspective article aims to delineate the conceptual basis of the “risk-integration” paradigm, outline a practical framework built on the pillars of precision assessment, dynamic monitoring, and coordinated intervention, as visually illustrated in Figure 2, which depicts how this integrated logic unfolds throughout the patient journey, and discuss potential implementation pathways. Our objective is to provide a coherent and clinically actionable strategy for navigating this longstanding challenge in cardiovascular care.

## 2 Limitations of the “risk-confrontation” paradigm and the need for reflection

The conventional “risk-confrontation” paradigm frames ischemic and bleeding risks as fundamentally opposed (11). This perspective often leads to binary or alternating therapeutic approaches—prioritizing one risk at the expense of the other (11, 12). For example, a bleeding event typically triggers a reduction or cessation of antithrombotic therapy, whereas subsequent ischemia prompts its reintensification (13). This reactive, oscillating strategy fails to address the underlying complexity of managing concurrent risks, resulting in fragmented and inconsistent care (14).

Several key limitations arise from this model. First, it promotes therapeutic instability, as clinical decisions become reactive rather than proactive, causing treatment plans to fluctuate with each adverse event (15). Second, risk assessment remains static and compartmentalized. Tools such as PRECISE-DAPT or HAS-BLED are often applied in isolation at baseline and are rarely updated, overlooking how risk evolves dynamically over time and how shared risk factors—such as renal impairment or advanced age—amplify both bleeding and ischemic susceptibility (14). Third, the paradigm encourages generalized treatment approaches that fail to account for clinically important distinctions, such as differences in bleeding etiology (e.g., ulcer-related vs. vascular malformation-related) or severity, leading to suboptimal management (16). Finally, it often excludes patient perspectives—personal values, preferences, and tolerance for specific risks are seldom integrated, reducing the acceptability and sustainability of treatment plans (17).

In summary, the “risk-confrontation” model, characterized by reactive decision-making, siloed risk evaluation, and uniform therapeutic responses, proves inadequate for patients with CAD at risk of GIB (18). Its shortcomings underscore the necessity of shifting toward an integrated approach that simultaneously evaluates, monitors, and addresses both ischemic and bleeding risks within a unified, patient-centered framework (19) (Supplementary Table 1).

## 3 Theoretical framework and core pillars of the “risk-integration” paradigm

The “risk-integration” paradigm moves beyond the traditional trade-off model, framing a patient's ischemic and bleeding risks, comorbidities, pathophysiology, and psychosocial factors as a dynamic, interconnected system (Supplementary Table 1). The therapeutic objective is not simply to minimize any single risk, but to optimize overall net clinical benefit throughout the course of care, balancing potential harms against benefits. This framework is supported by four interdependent components. The structural interaction among these pillars is schematically illustrated in Figure 2 to enhance conceptual readability.

### 3.1 First pillar: dynamic, multidimensional, and integrated risk assessment

This moves beyond static baseline scores by incorporating serially measured biomarkers (e.g., growth differentiation factor-15), repeated imaging or endoscopic findings, and evolving clinical parameters such as renal function (20, 21). The forward-looking aim is to develop integrated predictive tools capable of generating a unified risk profile that simultaneously reflects both ischemic and bleeding probabilities, or ideally, a quantifiable net-benefit index to guide therapeutic intensity with greater precision (22).

### 3.2 Second pillar: pathophysiology-guided precision in antithrombotic therapy and gastrointestinal protection

Antithrombotic management should be refined through strategies such as P2Y12 inhibitor selection guided by platelet function or CYP2C19 genotyping, individualized balancing of dual antiplatelet therapy duration vs. dual-pathway inhibition, and clear positioning of newer antithrombotic agents (23, 24). Gastrointestinal protection should be equally precise and integrated, involving routine Helicobacter pylori screening and eradication, etiology-specific use of proton-pump inhibitors (e.g., differentiating ulcer- from vascular-related bleeding), adjunctive mucosal protectants, and pre-emptive endoscopic evaluation in high-risk patients (25, 26).

### 3.3 Third pillar: time-phased and personalized treatment roadmaps

Clinical management should be structured according to distinct phases—acute (e.g., active bleeding or post-PCI), stabilization, and long-term maintenance—each with defined priorities (27, 28). Crucially, an individualized “contingency plan” should be pre-established based on the patient's specific risk profile, outlining clear steps for adjusting, holding, or resuming medications in scenarios such as minor bleeding or planned invasive procedures, thus minimizing reactive and inconsistent decision-making (8, 29).

### 3.4 Fourth pillar: patient-centered shared decision-making

This pillar formally integrates patient values, preferences, and life goals into the clinical pathway. Through structured decision aids, patients are enabled to visualize the comparative probabilities and consequences of ischemic vs. bleeding events (30, 31). On this foundation, clinicians and patients collaboratively define personalized treatment aims—for instance, whether to prioritize stroke prevention or to place greater emphasis on avoiding bleeding-related morbidity—thereby fostering adherence and aligning management with individual priorities (30, 32).

### 3.5 Operational algorithm for translating integrated risk assessment into practice

To improve practical application, we propose an operational algorithm that translates integrated risk principles into a structured clinical workflow.

Step 1: Comprehensive baseline assessment. Clinicians compile a unified patient profile including ischemic history, bleeding phenotype, comorbidities, frailty, renal function, endoscopic findings, and patient preferences.
Step 2: Identification of primary risk. Patients are classified as ischemia-dominant, bleeding-dominant, or mixed-risk. This classification guides the initial intensity of antithrombotic and gastroprotective therapies.
Step 3: Pre-defined contingency planning. Management pathways for foreseeable events—such as a decline in hemoglobin, minor bleeding, or planned procedures—are established in advance to avoid abrupt treatment changes.
Step 4: Scheduled dynamic reassessment. Risk is reevaluated at clinically relevant intervals or after significant clinical events, with therapy adjusted according to updated net clinical benefit.
Step 5: Shared decision-making reconfirmation. The treatment plan is reviewed with the patient to ensure alignment with evolving preferences and tolerance for uncertainty.

This algorithm shifts care from reactive intervention to coordinated longitudinal management, supporting consistent and multidisciplinary implementation.

Together, these pillars form a cohesive, proactive, and systematic framework designed to deliver truly individualized care for patients with coronary artery disease who are at concurrent risk of bleeding.

## 4 Pathways and challenges in implementing the new paradigm

The successful implementation of the “risk-integration” paradigm requires deliberate structural and operational changes in clinical practice, alongside an honest appraisal of existing systemic barriers.

A fundamental requirement is the establishment of a functioning, standing multidisciplinary team (33). This team-including cardiologists, gastroenterologists, hematologists, clinical pharmacists, and primary care physicians-should operate through scheduled collaboration, not episodic consultation (10, 34, 35). Its role is to jointly perform continuous risk assessment, co-design personalized treatment plans, and ensure consistent follow-through across care transitions (36). The operational algorithm establishes a standardized framework for such multidisciplinary collaboration, defining clear criteria for therapeutic reassessment and adjustment, which minimizes specialty-specific variability. Furthermore, the algorithm facilitates seamless care continuity between inpatient and outpatient settings, reinforcing this collaborative structure.

Technology and data infrastructure are essential enablers. Electronic health records should be designed to automatically aggregate key clinical variables—such as serial biomarker trends, endoscopic findings, and renal function—into a unified, dynamic risk profile (22, 37). Beyond documentation, artificial intelligence systems could further analyze this integrated data to generate patient-specific management suggestions, supporting rather than replacing clinical judgment (38, 39).

To build the necessary evidence base, clinical research should evolve. Priority should be given to precision-medicine studies that stratify patients by phenotype or endotype, identifying subgroups most likely to benefit from specific integrated strategies (40, 41). Pragmatic trials comparing holistic management protocols against usual care in real-world settings are equally important to demonstrate practical effectiveness and net clinical benefit (42, 43).

Significant challenges remain. First, there is a paucity of high-level evidence directly supporting integrated management, as most existing guidelines are founded on a compartmentalized view of risk (44). Second, healthcare system fragmentation—including specialty silos, discontinuous care pathways, and reimbursement models that favor episodic over coordinated care—hinders implementation (45). Third, clinician mindset and training should advance from applying isolated risk scores toward systems-based thinking and shared decision-making (46). Finally, rigorous health economic analyses are needed to evaluate whether the upfront investment in multidisciplinary care and monitoring is offset by long-term reductions in adverse events and hospitalizations (47).

Collectively, the shift from “risk confrontation” to “risk integration” represents a necessary evolution in managing patients with coronary artery disease and gastrointestinal bleeding risk (48, 49). This paradigm reconceptualizes the patient as a dynamic risk system, aiming to optimize long-term outcomes through integrated assessment, mechanism-based therapy, staged management, and collaborative goal-setting (50, 51). While substantial challenges—evidentiary, structural, educational, and economic—should be addressed, this framework provides a coherent and patient-centered path forward (52). We urge the clinical and research communities to prioritize the development of practical tools, interdisciplinary care models, and robust studies that will allow this integrative approach to benefit patients in real-world practice (48).

## 5 Summary

The transition from a “risk-confrontation” to a “risk-integration” paradigm represents a necessary evolution in the management of patients with coronary artery disease who are at risk of gastrointestinal bleeding. This framework redefines clinical decision-making by adopting a systemic perspective, which emphasizes dynamic risk evaluation, precision in therapeutic intervention, and the active inclusion of patient preferences. With the addition of a clear workflow algorithm and visual structure, the framework can be directly applied in routine clinical practice.

Moving forward, advancing this approach will depend on collaborative efforts across academic, clinical, and healthcare-administrative domains. Priorities should include the development of integrated risk-assessment tools, wider implementation of structured multidisciplinary care pathways, prospective research focused on personalized treatment strategies, and systematic redesign of clinical workflows. Through such coordinated initiatives, it will be possible to establish an individualized, outcome-oriented model of antithrombotic care that is both clinically effective and sustainable for this high-risk patient population.

## Data Availability

The original contributions presented in the study are included in the article/Supplementary Material, further inquiries can be directed to the corresponding author/s.
