# Supplementary material for: Toward an integrated risk paradigm: personalizing antithrombotic therapy for coronary heart disease with gastrointestinal bleeding
Source: Front Cardiovasc Med. 2026 Apr 14;13:1782409. doi: 10.3389/fcvm.2026.1782409 (PMC13121291; doi:10.3389/fcvm.2026.1782409)
Supplement: Supplementary file 1 [file Table1.docx]

**Supplementary Table 1. Comparison between risk-confrontation and risk-integration paradigms in managing coronary artery disease with gastrointestinal bleeding**

| Dimension | Risk Confrontation | Risk Integration | Clinical Implication |
| --- | --- | --- | --- |
| Risk assessment | Separate, static scores | Integrated, dynamic profile | Continuous adjustment |
| Therapeutic logic | Trade-off between risks | Net clinical benefit | More stable strategy |
| Treatment timing | Reactive | Phase-specific | Fewer abrupt  interruptions |
| GI protection | Empirical PPI use | Etiology-guided prevention | Lower recurrent  bleeding |
| Decision-making | Physician-driven | Shared decision-making | Better adherence |
| Care model | Specialty silos | MDT-based coordination | Continuity of care |

**Abbreviations**: GI, gastrointestinal; MDT, multidisciplinary team; PPI, proton pump inhibitor.
